# Ectopic Gestation

**Published:** 1899-02-25

**Authors:** 


### ECTOPIC GESTATION.

At a recent meeting of the Obstetrical Society of London Dr. Archibald Donald read a paper on a case of Ectopic Gestation, in which the fœtus was extracted at the seventh month by vaginal incision. The abdomen was uniformly enlarged by a tumour, which reached about four inches above the umbilicus. A loud *souffle* was heard. The uterus when examined bimanually was found to be about the size of a two-months' pregnant uterus. The cervix was close to the pubes, and the posterior fornix was bulged by the fœtal head, the sutures in which could be felt. Abdominal section was performed, and the placenta was exposed. The wound was then temporarily closed, and the child delivered through a posterior vaginal incision by cranioclasm, the sac being packed with iodoform gauze. On the removal of this gauze on the fourth day alarming hæmorrhage occurred. The separation of the placenta was not completed for some weeks. In commenting on this paper, Dr. Galabin pointed out the advantages of the preliminary abdominal incision. If on this being done the case proved to be one of intra-ligamentous pregnancy unruptured, or if the pelvis was completely shut off by adhesions, the sac could be opened *per vaginam;* on the other hand, if, as had happened to him in one case, it should turn out that the sac had ruptured, and that there was effusion of blood into the peritoneal cavity, this condition could be dealt with.

